# Influences of volcano eruptions on Asian **S**ummer Monsoon over the last 110 years

**DOI:** 10.1038/srep42626

**Published:** 2017-02-16

**Authors:** Liang Ning, Jian Liu, Weiyi Sun

**Affiliations:** 1 Key Laboratory of Virtual Geographic Environment, Ministry of Education; State key Laboratory of Geographical Environment Evolution, Jiangsu Provincial Cultivation Base; School of Geography Science, Nanjing Normal University, Nanjing, 210023, China; 2 Jiangsu Center for Collaborative Innovation in Geographical Information Resource Development and Application, Nanjing, 210023, China; 3 Climate System Research Center, Department of Geosciences, University of Massachusetts, Amherst, 01003, United States

## Abstract

Asian summer monsoon (ASM) precipitation is the primary water resource for agriculture in many Asian countries that have experienced rapid economic growth in recent decades, thus implying the necessity for further investigations on both the internal variability of the ASM and the influence of external factors on the ASM. Using long-term high-resolution (0.5° × 0.5°) observed precipitation data, contrary to previous studies on inter-annual timescale, we showed that over the last 110 years, volcanic eruptions have influenced ASM variations on an inter-decadal timescale via teleconnections with the Atlantic Multi-decadal Oscillation (AMO). This relationship was also confirmed by Coupled Model Intercomparison Program Phase 5 (CMIP5) model simulations. During the active volcanic eruption periods (1901–1935 and 1963–1993), significantly lower ASM precipitation was observed compared with that during the inactive volcanic eruption period (1936–1962). We found that during active volcanic eruption periods, which correspond to a negative AMO state, there is an anomalously weakened Walker circulation over the tropical Pacific that transports less moisture to the ASM region and subsequently reduces ASM precipitation. This new finding may help improve decadal predictions of future changes in the ASM.

The demand for decadal climate predictions is currently increasing, which is necessary for infrastructure planning, energy policies, economic development, and societal sustainability^1^. Such predictions are especially important for the region affected by the Asian summer monsoon (ASM), which influences more than 50% of the world’s population^2^,^3^. Opposite trends in the ASM have been observed in different periods over the last six decades^4^,^5^,^6^, indicating that ASM variability and the corresponding mechanisms should be investigated over a longer time period. In addition to its own internal variability, the ASM variability is also influenced by external forcings, including natural forcing (e.g., solar radiation and volcanic eruptions) and anthropogenic forcing (e.g., greenhouse gas emissions and land use/land cover change)^7^,^8^.

Regarding the influence of volcanic eruptions^9^,^10^, previous studies have shown that large volcanic eruptions affect summer monsoon precipitation on global and regional scales^11^,^12^,^13^. Over the Asian monsoon region, large volcanic eruptions correspond to a weakening of the East Asian Summer Monsoon (EASM) circulation and subsequent reductions in EASM rainfall^14^,^15^,^16^,^17^ although the impacts are relatively short lived^11^.

However, on the aspect of long-term influence, both proxy records^18^ and model simulations^19^ suggest that volcanic eruptions have played a dominant role in pacing the Atlantic Multi-decadal Oscillation (AMO), which has a potential influence on the EASM^3^,^20^, since the end of the Little Ice Age. These findings highlight the potential multi-decadal influences of large volcanic eruptions on the ASM through teleconnections with the AMO. Because the AMO is believed to be driven by thermohaline circulation^21^ and may be predictable, developing a better understanding of the influence of volcanic eruptions on the ASM through the AMO will help to improve decadal predictions of future ASM changes.

## Influence of volcanic eruptions on the ASM over inter-decadal timescales

The Asian monsoon domain (Fig. S1) is defined by the regions in which the difference between summer precipitation (June-July-August, JJA) and winter precipitation (December-January-February, DJF) exceeds 180 mm and the summer monsoon precipitation exceeds 35% of the total annual precipitation^4^. This definition has been used widely in the study of monsoon variability^5^.

**Figure 1:**
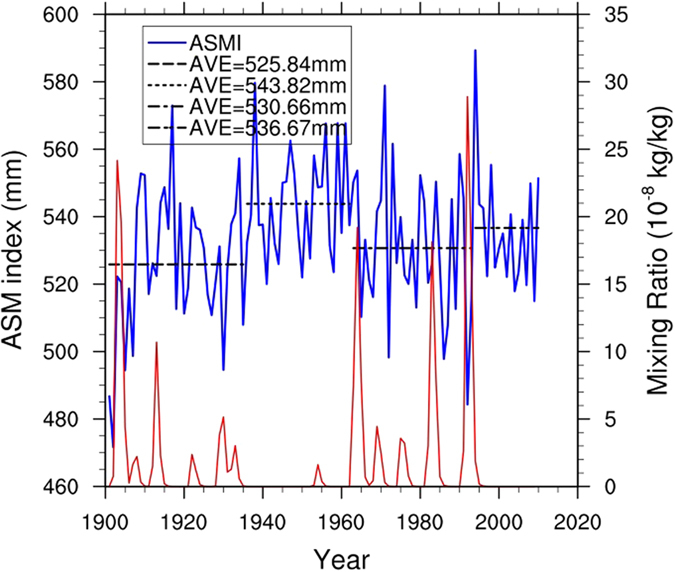
The time series of observed Asian summer monsoon index (blue solid line; left y-axis; unit: mm) and reconstructed volcanic aerosol mass mixing ratio (red solid line; right y-axis; unit: 10^−8^ kg/kg) over the period 1901–2010.

We selected two periods based on the number of volcanic eruptions with volcanic aerosol mixing ratios larger than 2 × 10^−8^ kg/kg (1901–1935 and 1963–1993) and compared these with the intervening period (1936–1962) in which volcanic activity was minimal^22^. Figure 1 shows that during the active volcanic eruption periods, the averages of the ASM index (defined as the total JJA precipitation averaged in the Asian monsoon domain^4^,^5^) were 525.8 mm and 530.7 mm, and both of these values were significantly lower than the ASM index of 543.8 mm during the inactive volcanic eruption period 1936–1962 based on Student’s t-test (*p* < 0.05). This inter-decadal scale variation is different from the short-term influences of volcanic eruptions on the ASM index found in previous studies^11^. Inter-decadal variability can also explain the different trends over the period since the 1950s^4^,^5^ and the period since the 1980s^6^.

To confirm that this inter-decadal variability was induced by the volcanic eruptions, a total of four simulations were run using the Community Earth System Model version 1 with Community Atmospheric Model version 5 (CESM1-CAM5) from the Coupled Model Intercomparison Program Phase 5 (CMIP5) to differentiate the influences from anthropogenic forcing (i.e., anthropogenic greenhouse gas, anthropogenic aerosol, and land use and cover change), solar radiation forcing, and volcanic forcing. These four simulations included one simulation forced by all three forcing (anthropogenic forcing, solar radiation forcing, and volcanic forcing) and three sensitivity experiments forced by individual forcing over the period from 1901 to 2005. In the all-forcing experiment, the simulated major characteristic of the ASM was a linear decreasing trend over the entire period (Fig. S2a), which was mainly induced by the anthropogenic forcing (Fig. S2b). However, the simulated ASM in the solar radiation forcing sensitivity experiment did not show significant inter-decadal variability (Fig. S2c), indicating the solar radiation did not induce ASM inter-decadal variability over the last century. The only inter-decadal variability similar to the observation was from the volcanic forcing sensitivity experiment, especially the significant decrease of ASM precipitation for the period from 1963 to 1993 (Fig. S2d). The simulated decrease of the ASM for the period from 1901 to 1935 is not obvious because the decrease in 1930 was not significant (Fig. S2e), which indicated that the model is not sensitive to small volcanic eruptions. Among these four simulations, the observed inter-decadal variability of the ASM was only reproduced by the sensitivity experiment of volcanic forcing. Therefore, a comparison among the model simulations confirms that volcanic forcing is the major contributor to the inter-decadal variability of ASM.

When the large-scale modes of climate variability were considered (Fig. S3), the total ASM index was significantly correlated with the AMO index (r = 0.21, *p* < 0.05). During warm (cold) AMO phases, there were positive (negative) ASM indices, and similar relationships were also observed in previous studies^3^,^20^.

**Figure 2:**
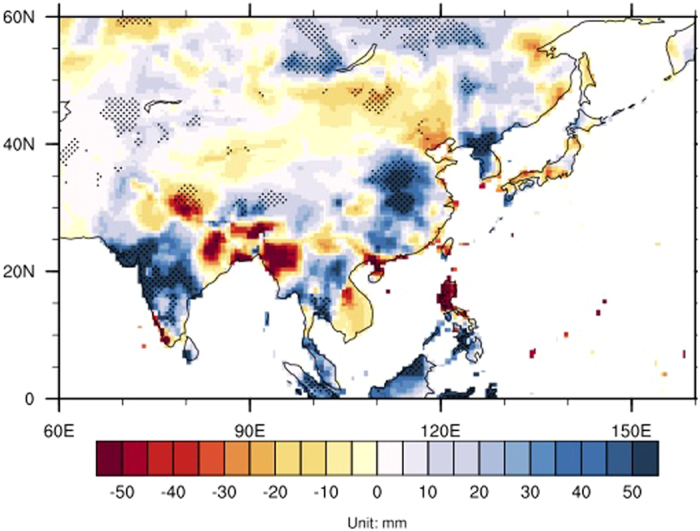
The regression of AMO index on the Asian summer monsoon precipitation (unit: mm) over the period 1901–2010. Stippling indicates the correlations are significant at the *p* < 0.1 level. Map was generated by NCAR Command Language (NCL). The NCAR Command Language (Version 6.3.0) [Software]. (2016). Boulder, Colorado: UCAR/NCAR/CISL/TDD. http://dx.doi.org/10.5065/D6WD3XH5.

This relationship was further confirmed by regressions of the AMO index on the ASM precipitation over the Asian monsoon region (Fig. 2). Significant positive precipitation anomalies (~30–50 mm) were observed over large parts of the Asian monsoon region, including South Asia, East Asia, and Northeast Asia. The results show that a positive AMO significantly increases ASM precipitation.

To investigate the influence of volcanic eruptions on AMO variability, differences in the detrended winter and summer sea surface temperature (SST) between the inactive volcanic eruption period (1936–1962) and active volcanic eruption periods (1901–1935 and 1963–1993) were assessed and are shown in Fig. S4. During the inactive volcanic eruption periods, the SSTs were significantly higher over the entire northern Atlantic than they were in the active volcanic eruption periods, and the magnitude of the difference was 0.2–0.6 °C. The variance of AMO index from the pre-industrial control experiment (PIControl) and the volcanic forcing sensitivity experiment were also compared. The results showed that the differences between the standard deviations of the AMO in PIcontrol and in Vol experiment were not significant based on the f-test. Whereas, during the active volcanic eruption periods (1963–1993), the AMO were significantly lower than the AMO during the inactive volcanic eruption periods (1936–1962) based on Student’s t-test (*p* < 0.05) (Fig. S5). These suggested that the volcanic forcing may not significantly influence the variance of the AMO, but it significantly decreased the magnitude of the AMO. Proxy records also suggest that external forcing played a dominant role in pacing the AMO during the period after the Little Ice Age, and an instantaneous impact was observed on mid-latitude SST that spread across the North Atlantic over the ensuing ~5 years through the Atlantic Meridional Overturning Circulation (AMOC)^18^. Model results also show that aerosol emissions and periods of volcanic activity explain 76% of the simulated multi-decadal variance in the detrended 1860–2005 North Atlantic SST^19^. Moreover, significant SST warming was observed over the northern Pacific along the coast and in the eastern subtropical Pacific, whereas the SST anomalies are slightly negative over the eastern tropical Pacific, which is similar to a La Niña condition.

In addition to the influence on the AMO from volcanic forcing, the influence of the AMOC was also investigated by comparing lag correlations between low-pass filtered AMOC and AMO indices from the PIControl, the all-forcing experiment, and the volcanic forcing sensitivity experiment. Without external forcing, negative correlations were observed between the AMOC and AMO, with the AMOC leading by approximately 10 years (Fig. S6), thus indicating that the AMOC has an influence on regulating the Atlantic SST. These negative correlations were enhanced in the all-forcing experiment and volcanic forcing sensitivity experiment, which showed significantly negative and nearly simultaneous correlations. This finding indicated that the timing and magnitude of the internal influence on the AMO from the AMOC can also be changed by external forcing^23^,^24^.

## Mechanisms underlying the influence of the AMO on ASM precipitation

**Figure 3:**
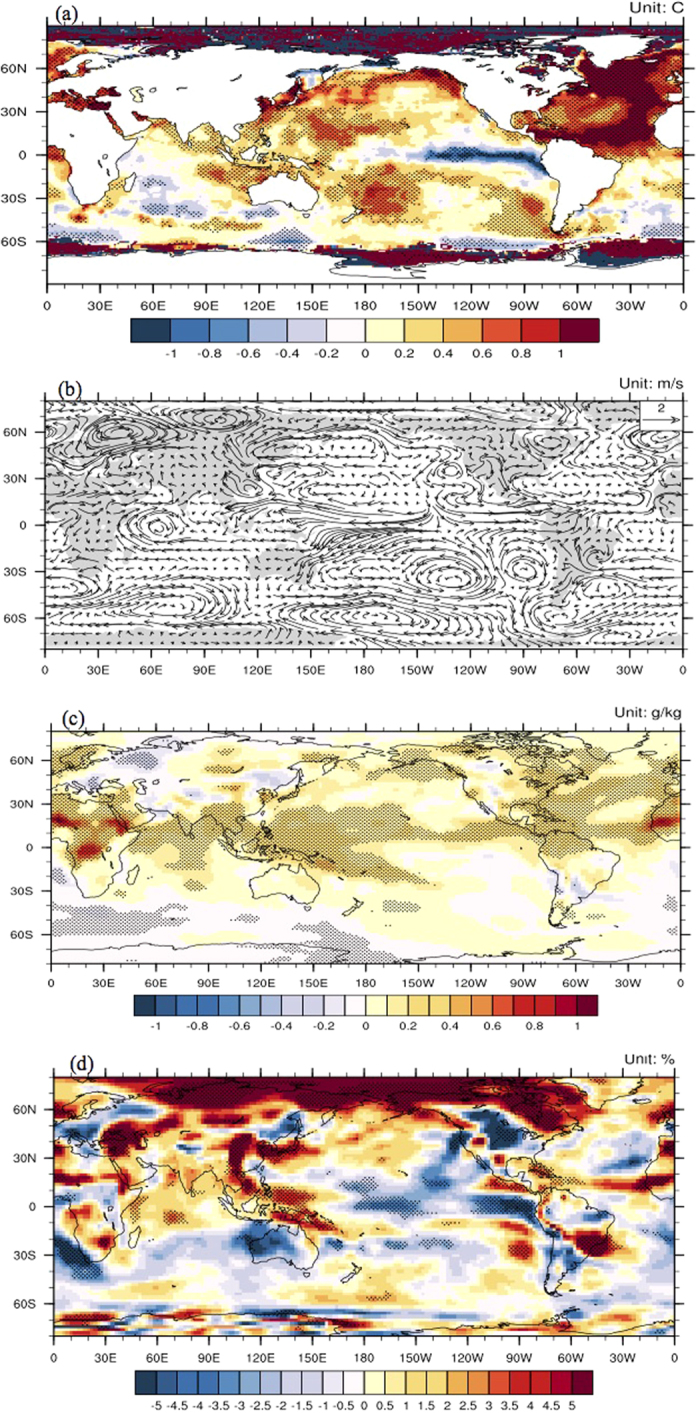
The regression of AMO index on summer detrended SST (**a**, unit: °C), 850hPa wind (**b**, unit: m/s), detrended specific humidity (**c**, unit: g/kg), and relative humidity (**d**, unit: %) over the period 1901–2010Stippling indicates the correlations are significant at the *p* = 0.05 level. Maps were generated by NCAR Command Language (NCL). The NCAR Command Language (Version 6.3.0) [Software]. (2016). Boulder, Colorado: UCAR/NCAR/CISL/TDD. http://dx.doi.org/10.5065/D6WD3XH5.

The regression of the AMO on the global JJA SST showed a more obvious influence over the Atlantic as well as the Pacific, and significant SST warming was observed over the western Pacific and significant SST cooling was observed over the eastern tropical Pacific (Fig. 3a). Although the reconstruction data^25^,^26^ and model evidence^27^ revealed a robust El Niño Southern Oscillation (ENSO) response to large tropical eruptions on an inter-annual scale over the past several centuries, the results here indicate that volcanic eruptions also have similar influences on the inter-decadal timescale because of amplification through the AMO. Notably, the correlation coefficient between the AMO index and the Nino3.4 index was insignificant on inter-annual timescales^3^, and significant differences were not observed in the occurrences of El Niño events during the positive and negative AMO periods. Therefore, this inter-decadal influence of volcanic eruptions on the eastern tropical Pacific SST through the AMO is independent of the influences of El Niño events on inter-annual scales as observed in previous studies^25^,^26^,^27^.

The AMO is a significant driver of climate over the whole Northern Hemisphere. The warming in the North Atlantic corresponds to suppressed rainfall in the tropical central Pacific, easterly anomalies over the western Pacific and westerly anomalies over the rest of the northern tropics, which enhance the Northern Hemisphere Summer Monsoon (NHSM)^3^. The NHSM system, which includes the ASM, is highly influenced by both the Hadley and the Walker circulations^28^. During periods with a positive AMO index, the corresponding mechanism underlying the influences of the AMO on the ASM precipitation include the strengthening of the Walker circulation over the western tropical Pacific as well as anomalous westerlies and corresponding southerlies over the Asian monsoon region (Fig. 3b). The detailed circulation pattern over the Asian monsoon region is shown in Fig. S7. This circulation pattern enhances water vapor transport to the Asian monsoon region from the western Pacific Ocean and Indian Ocean, which results in increased specific humidity (Fig. 3c) and relative humidity (Fig. 3d). These increases are crucial to the Asia Monsoon rainfall^29^; therefore, additional ASM precipitation occurs during periods with a positive AMO index. Moreover, volcanic forcing can also affect Asian summer monsoon directly by changing the thermal contrast between the Eurasian continent and the adjacent oceans. To explore this effect, an index of land-sea thermal contrast over the ASM region has been defined as the standardized JJA surface temperature difference between the Eurasian continent (10°–60°N, 70°–140°E) and the adjacent oceans (20°S–50°N, 60°–160°E) in Vol experiment. The results showed that the land-sea thermal contrast decreased during active volcanic eruption periods, but the differences were not significant based on Student’s t-test (Fig. S8). Moreover, the mechanism that land-sea thermal contrast influences the ASM is different from the AMO mechanism (Fig. S9). Therefore, it can be concluded that the volcanic forcing’s direct effect by changing the thermal contrast has a contribution to decrease the ASM during the active volcanic eruption periods, but it is not significant.

These results demonstrate that volcanic eruption-induced AMO-related Atlantic SST anomalies have robust influences on the SST pattern and Walker circulation over the tropical Pacific as well as on the corresponding circulation and monsoon precipitation over the Asian monsoon region. During the inactive volcanic eruption period, positive SST anomalies were observed over the northern Atlantic and the western tropical Pacific but negative SST anomalies were observed over the eastern Pacific, and the corresponding enhanced Walker circulation transported additional water vapor to the ASM region and increased monsoon precipitation. The results presented here show that volcanic eruptions have a strong influence on ASM precipitation via teleconnections with the AMO, and this finding has important implications for improving future predictions of ASM changes on the decadal timescale, which will help resource managers and decision makers prepare corresponding adaptation plans for future sustainable economic development.

## Methods

To investigate the long-term variability of the ASM over the last 110 years, the high-resolution (0.5° × 0.5°) monthly observed precipitation data^30^ from the University of East Anglia Climatic Research Unit (CRU) TS v.3.22 were used to define the ASM index. The long-term AMO index (unsmoothed), Nino3.4 index and the National Centers for Environmental Prediction (NCEP) reanalysis data were downloaded from the National Oceanic & Atmospheric Administration (NOAA) Earth System Research Laboratory (ERSL) website (http://www.esrl.noaa.gov/psd/data/). The AMO index was calculated over the North Atlantic (0°–70°N) using the Kaplan SST data^31^, and the Nino3.4 index was calculated over the east central tropical Pacific (5°N–5°S, 170°–120°W). The volcanic aerosol data were reconstructed using proxy data (Ammann *et al*.)^22^. The Hadley Centre Sea Ice and Sea Surface Temperature data set (HadISST) was obtained from the U.K. Meteorological Office^32^.

Linear correlation and linear regression methods were applied to examine the relationships between the AMO and ASM precipitation, AMO influences on SSTs, low-level winds, relative humidity, and specific humidity. To examine the inter-decadal variability of SSTs and relative humidity, the linear trends calculated by the ordinary least squares method were subtracted first to remove the influences from anthropogenic GHG emissions.

The AMO indices were defined as the mean SST averaged over the region (0–60°N, 75–7.5°W), and the AMOC indices were defined as the maximum of the stream function over the northern Atlantic (20–50°N).

## Additional Information

**How to cite this article**: Ning, L. *et al*. Influences of volcano eruptions on Asian Summer Monsoon over the last 110 years. *Sci. Rep.* **7**, 42626; doi: 10.1038/srep42626 (2017).

**Publisher's note:** Springer Nature remains neutral with regard to jurisdictional claims in published maps and institutional affiliations.

## Supplementary Material

Supplementary Materials

## References

[b1] Meehl G. A. . Decadal prediction: Can it be skillful? Bull. Am. Meteorol. Soc. 90, 1467–1485 (2009).

[b2] Parthasarathy B., Diaz H. F. & Eischeid J. K. Prediction of all-India summer monsoon rainfall with regional and large-scale parameters, J. Geophys. Res. 93, 5341–5350 (1988).

[b3] Wang B. . Northern Hemisphere summer monsoon intensified by mega-El Niño/southern oscillation and Atlantic multidecadal oscillation, Proc. Natl. Acad. Sci. 110, 5347–5352, doi: 10.1073/pnas.1219405110 (2013). 23509281 PMC3619285

[b4] Wang B. & Ding Q. Changes in global monsoon precipitation over the past 56 years, Geophy. Res. Lett. 33, L06711, doi: 10.1029/2005GL025347 (2006).

[b5] Zhou T., Zhang L. & Li H. Changes in global land monsoon area and total rainfall accumulation over the last half century, Geophy. Res. Lett. 35, L16707, doi: 10.1029/2008GL034881 (2008).

[b6] Wang B., Liu J., Kim H.-J., Webster P. J. & Yim S.-Y. Recent change of the global monsoon precipitation (1979–2008), Clim. Dyn. 39, 1123–1135 (2012).

[b7] Gao C., Robock A. & Ammann C. M. Volcanic forcing of climate over the past 1500 years: an improved ice core-based index for climate models. J. Geophys. Res. 113, D23111, doi: 10.1029/2008JD010239 (2008).

[b8] Ammann C. M. & Naveau P. A statistical volcanic forcing scenario generator for climate simulations. J. Geophys. Res. 115, D05107, doi: 10.1029/2009JD012550 (2010).

[b9] Ammann C. M., Meehl G. A., Washington W. M. & Zender C. S. A monthly and latitudinally varying volcanic forcing dataset in simulations of 20^th^ century climate. Geophy. Res. Lett. 30, 1657, doi: 10.1029/2003GL016875 (2003).

[b10] Bradley R. S. The explosive volcanic eruption signal in Northern Hemisphere continental temperature records. Climatic Change 12, 221–243 (1988).

[b11] Schneider D. P., Ammann C. M., Otto-Bliesner B. L. & Kaufman D. S. Climate response to large, high-latitude and low-latitude volcanic eruptions in the Community Climate System Model, J. Geophys. Res. 114, D15101, doi: 10.1029/2008JD011222 (2009).

[b12] Anchukaitis K. J. . Influence of volcanic eruptions on the climate of the Asian monsoon region, Geophy. Res. Lett. 37, L22703, doi: 10.1029/2010GL044843 (2010).

[b13] Man W., Zhou T. & Jungclaus J. H. Effects of large volcanic eruptions on global summer climate and East Asian Monsoon changes during the last millennium: analysis of MPI-ESM simulations, J. Climate 27, 7394–7409 (2014).

[b14] Fan F., Mann M. E. & Ammann C. M. Understanding changes in the Asian summer Monsoon over the past millennium: Insights from a long-term coupled model simulation, J. Climate 22, 1736–1748 (2009).

[b15] Iles C. E. & Hegerl G. C. The global precipitation response to volcanic eruptions in the CMIP5 models, Environ. Res. Lett. 9, doi: 10.1088/1748-9326/9/10/104012 (2014).

[b16] Man W. & Zhou T. Response of the East Asian summer monsoon to large volcanic eruptions during the last millennium, Chin. Sci. Bull. 59, 4123–4129 (2014).

[b17] Song F., Zhou T. & Qian Y. Responses of East Asian summer monsoon to natural and anthropogenic forcing in the 17 latest CMIP5 models, Geophy. Res. Lett. 41, doi: 10.1002/2013GL058705 (2014).

[b18] Knudsen M. F., Jacobsen B. H., Seidenkrantz M.-S. & Olsen J. Evidence for external forcing of the Atlantic Multidecadal Oscillation since termination of the Little Ice Age. Nature Communications 5, 3323, doi: 10.1038/ncomms4323 (2014). PMC3948066 24567051

[b19] Booth B. B. B., Dunstone N. J., Halloran P. R., Andrews T. & Bellouin N. Aerosols implicated as a prime driver of twentieth-century North Atlantic climate variability, Nature 484, 228–232 (2012). 22498628 10.1038/nature10946

[b20] Feng S. & Hu Q. How the North Atlantic Multidecadal Oscillation may have influenced the Indian summer monsoon during the past two millennia, Geophy. Res. Lett. 35, L01707, doi: 10.1029/2007GL032484 (2008).

[b21] Delworth T. L. & Mann M. E. Observed and simulated multidecadal variability in the Northern Hemisphere, Clim. Dyn. 16, 661–676 (2000).

[b22] Ammann C. M., Joos F., Schimel D. S., Otto-Bliesner B. L. & Tomas R. A. Solar influence on climate during the past millennium: Results from transient simulations with the NCAR Climate System Model, Proc. Natl. Acad. Sci. 104, 3713–3718 (2007). 17360418 10.1073/pnas.0605064103 PMC1810336

[b23] Ottera O. H., Bentsen M., Drange H. & Suo L. External forcing as a metronome for Atlantic multidecadal variability, Nature Geoscience 3, 688–694, doi: 10.1038/NGEO955 (2010).

[b24] Swingedouw D. . Bidecadal North Atlantic ocean circulation variability controlled by timing of volcanic eruptions, Nature Communications 6, 6545, doi: 10.1038/ncomms7545 (2015). 25818017

[b25] Adams J. B., Mann M. E. & Ammann C. M. Proxy evidence for an El Niño-like response to volcanic forcing, Nature 426, 274–278 (2003). 14628048 10.1038/nature02101

[b26] Li J. . El Niño modulations over the past seven centuries, Nature Climate Change 3, 822–826 (2013).

[b27] Mann M. E., Cane M. A., Zebiak S. E. & Clement A. Volcanic and solar forcing of the tropical Pacific over the past 1000 years, J. Climate 18, 447–456 (2005).

[b28] Webster P. J. . Monsoons: processes, predictability, and the prospects for prediction. J. Geophys. Res. 103, 14451–14510 (1998).

[b29] Zhou T. & Yu R. C. Atmospheric water vapor transport associated with typical anomalous summer rainfall patterns in China, J. Geophys. Res. 110, D08104, doi: 10.1029/2004JD005413 (2004).

[b30] Harris I., Jones P. D., Osborn T. J. & Lister D. H. Updated high-resolution grids of monthly climatic observations—the CRU TS3.10 Dataset. Int J Climatol 34, 623–642 (2014).

[b31] Enfield D. B., Mestas-Nunez A. M. & Trimble P. J. The Atlantic multidecadal oscillation and it’s relation to rainfall and river flows in the continental U.S. Geophy. Res. Lett. 28, 2077–2080 (2001).

[b32] Rayner N. A. . Global analysis of sea surface temperature, sea ice, and night marine air temperature since the late nineteenth century, J. Geophys. Res. 108, 4407, doi: 10.1029/2002JD002670 (2003).

