# Supplementary material for: Influences of volcano eruptions on Asian Summer Monsoon over the last 110 years
Source: Sci Rep. 2017 Feb 16;7:42626. doi: 10.1038/srep42626 (PMC5311988; doi:10.1038/srep42626)
Supplement: Supplementary Materials [file srep42626-s1.pdf]

Influences of volcano eruptions on Asian Summer Monsoon over the last 110 years

Liang Ning[1,2,3] , Jian Liu[1,2*], and Weiyi Sun[1,2]

[11]Key Laboratory of Virtual Geographic Environment, Ministry of Education; State key Laboratory of Geographical Environment Evolution, Jiangsu Provincial Cultivation Base; School of Geography Science, and Jiangsu Key Laboratory for Numerical Simulation of Large Scale Complex System, School of Mathematical Science, Nanjing Normal University, Nanjing, 210023, China

[2]Jiangsu Center for Collaborative Innovation in Geographical Information Resource Development and Application, Nanjing, 210023, China

[3]Climate System Research Center, Department of Geosciences, University of Massachusetts, Amherst, 01003, United States

[*]jliu@njnu.edu.cn

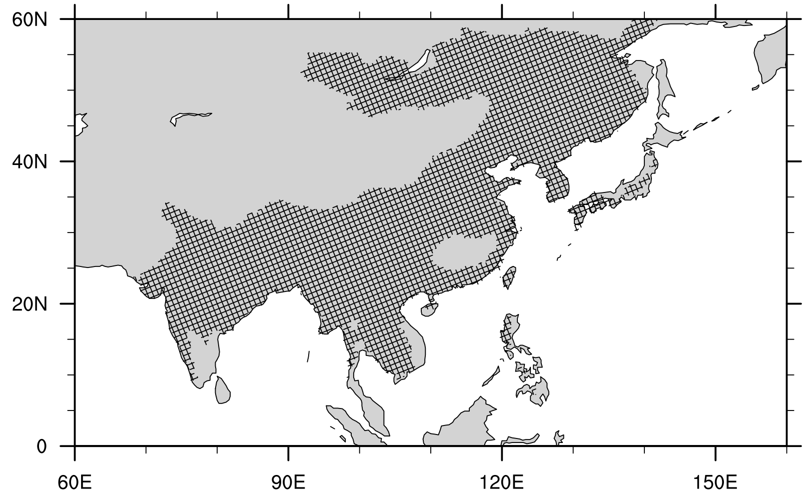

Fig. S1 Map of Asian monsoon domain (shaded area)

Map was generated by NCAR Command Language (NCL).

The NCAR Command Language (Version 6.3.0) [Software]. (2016).

Boulder, Colorado: UCAR/NCAR/CISL/TDD. http://dx.doi.org/10.5065/D6WD3XH5

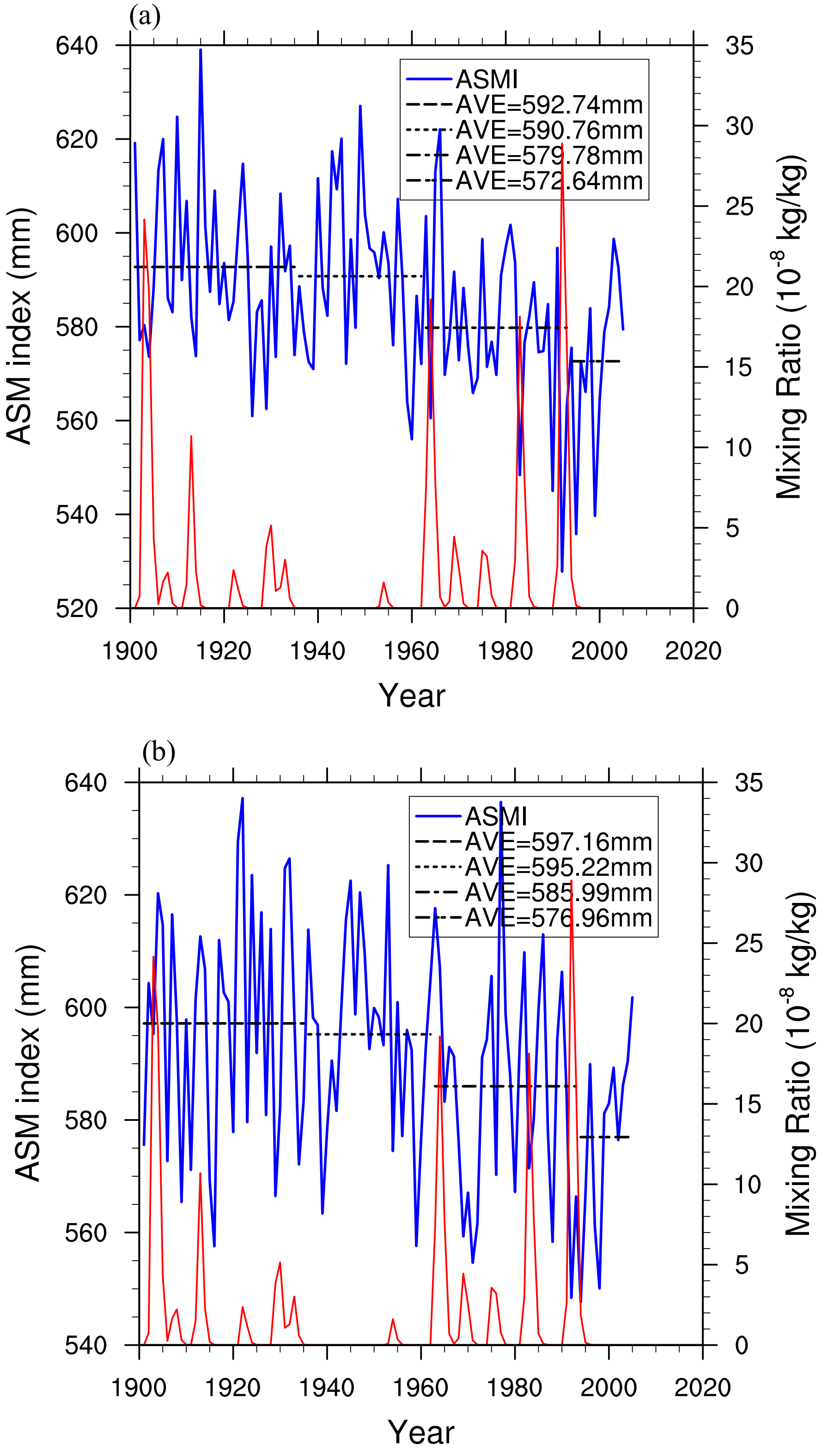
(a)
ASMI
AVE=592.74mm
AVE=590.76mm
AVE=579.78mm
AVE=572.64mm
ASM index (mm)
Mixing Ratio (10^{-8} kg/kg)
Year
640
620
600
580
560
540
520
35
30
25
20
15
10
5
0
1900
1920
1940
1960
1980
2000
2020
(b)
ASMI
AVE=597.16mm
AVE=595.22mm
AVE=585.99mm
AVE=576.96mm
ASM index (mm)
Mixing Ratio (10^{-8} kg/kg)
Year

(c)

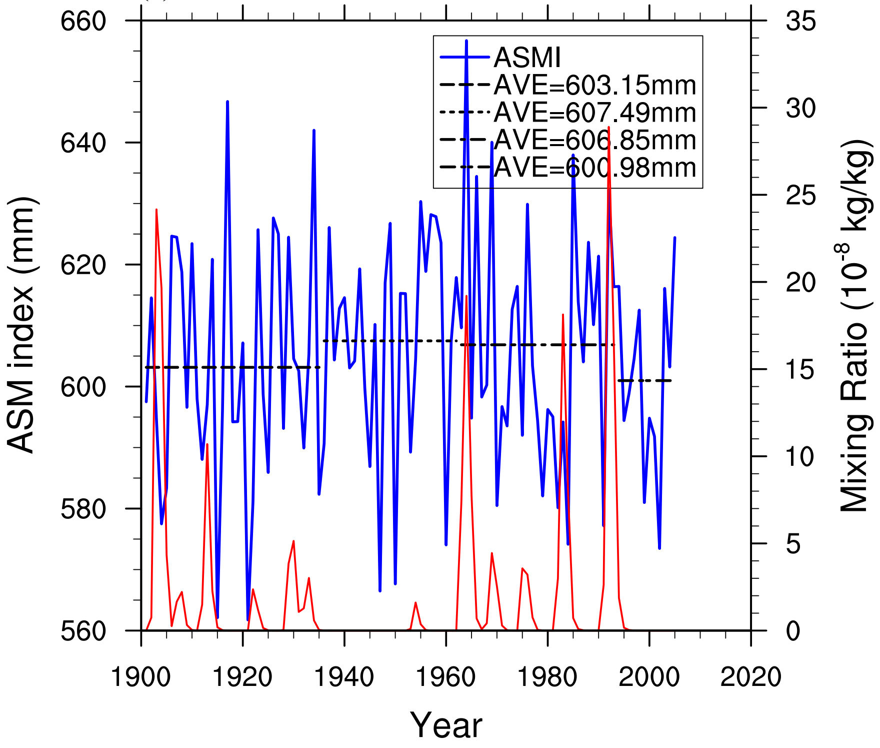
ASMI
AVE=603.15mm
AVE=607.49mm
AVE=606.85mm
AVE=600.98mm
ASM index (mm)
Mixing Ratio (10^{-8} kg/kg)
Year
660
640
620
600
580
560
35
30
25
20
15
10
5
0
1900
1920
1940
1960
1980
2000
2020

(d)

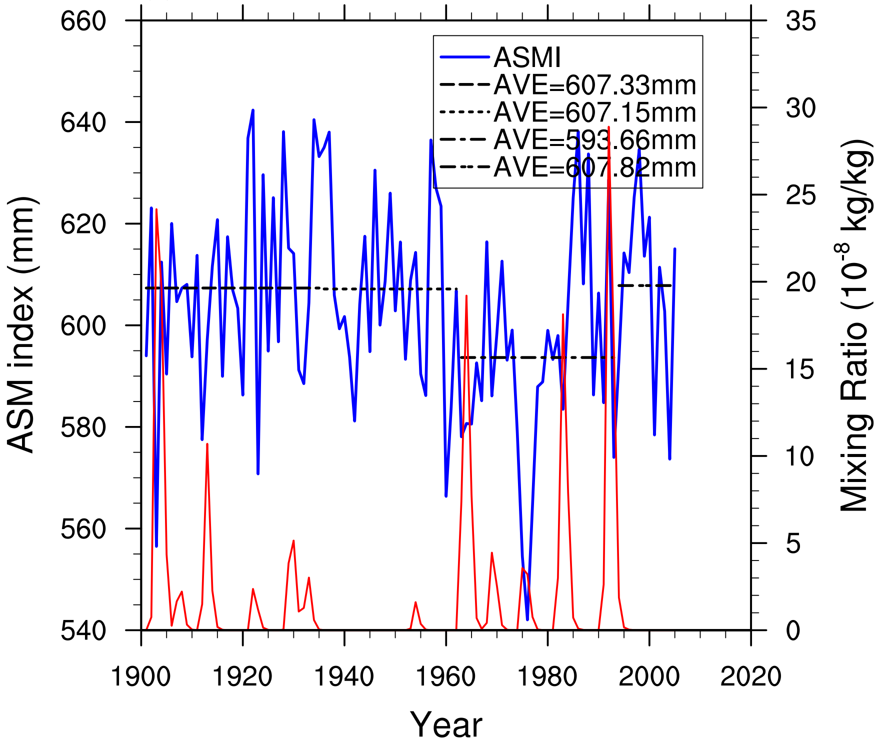
ASMI
AVE=607.33mm
AVE=607.15mm
AVE=593.66mm
AVE=607.82mm
ASM index (mm)
Mixing Ratio (10^{-8} kg/kg)
Year
660
640
620
600
580
560
540
35
30
25
20
15
10
5
0
1900
1920
1940
1960
1980
2000
2020

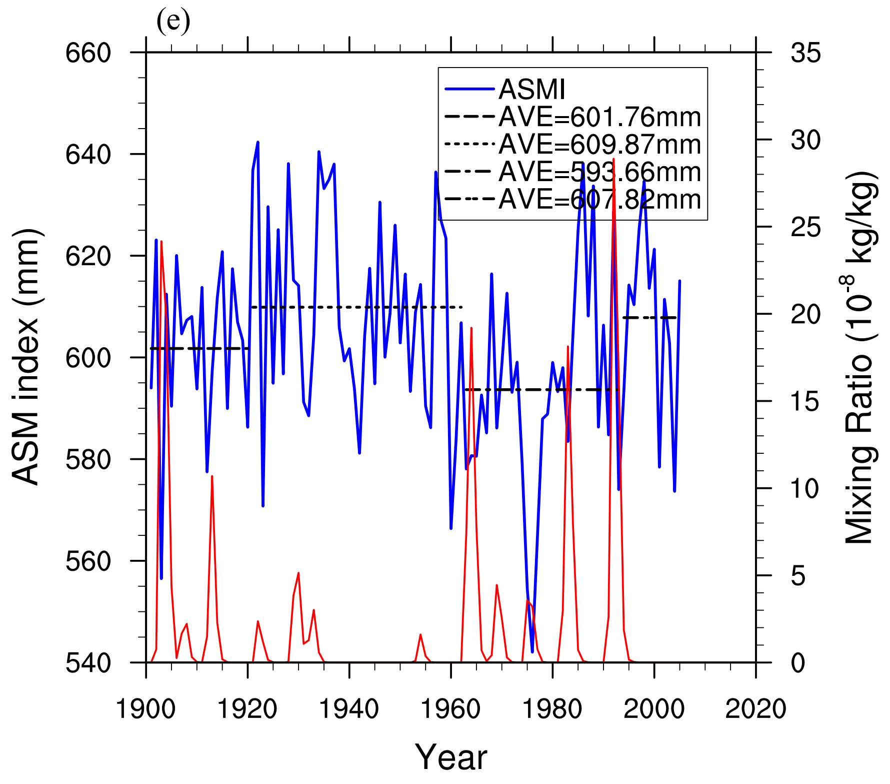

Fig. S2 The time series of simulated Asian summer monsoon index (blue solid line; left y-axis; unit: mm) and reconstructed volcanic aerosol mass mixing ratio (red solid line; right y-axis; unit: 10^{-8} kg/kg) from the CESM1-CAM5 all-forcing experiment (a), anthropogenic forcing sensitivity experiment (b), solar radiation forcing sensitivity experiment (c), and volcanic forcing sensitivity experiment (d &e) over the period 1901-2005

In Fig. S2e, the first active volcanic eruption period covers 1901-1920.

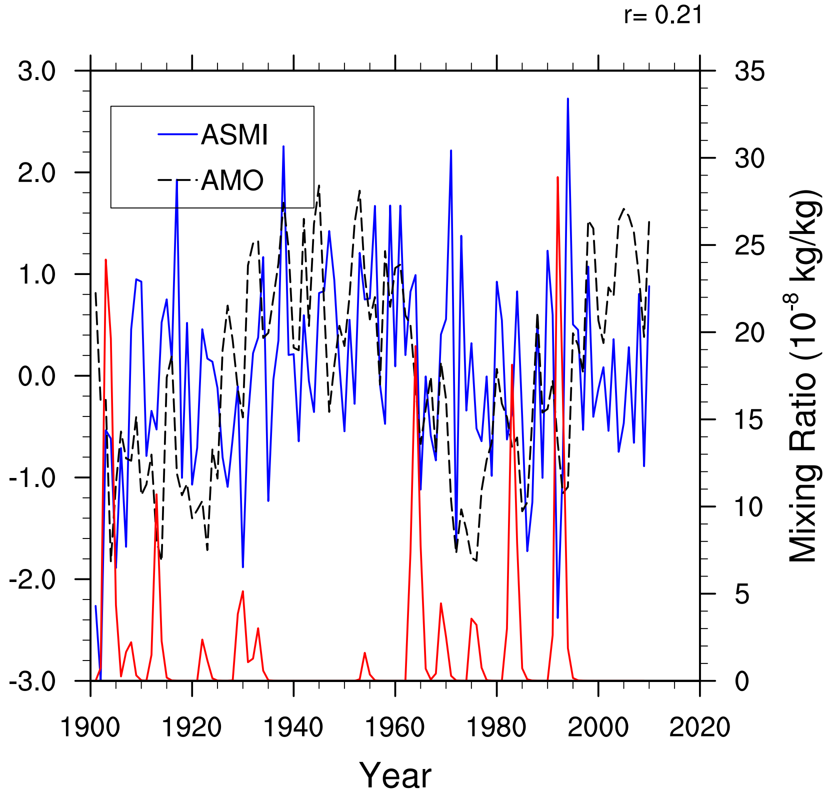

Fig. S3 The time series of standardized Asian summer monsoon index, standardized AMO index, and reconstructed volcanic aerosol mass mixing ratio over the period 1901-2010

The time series of standardized monsoon indices are shown as blue solid lines (left y-axis).

The time series of standardized AMO index are shown as black dash lines (left y-axis).

The time series of reconstructed volcanic aerosol mass mixing ratio are shown as red solid lines (right y-axis; unit: 10^{-8} kg/kg)

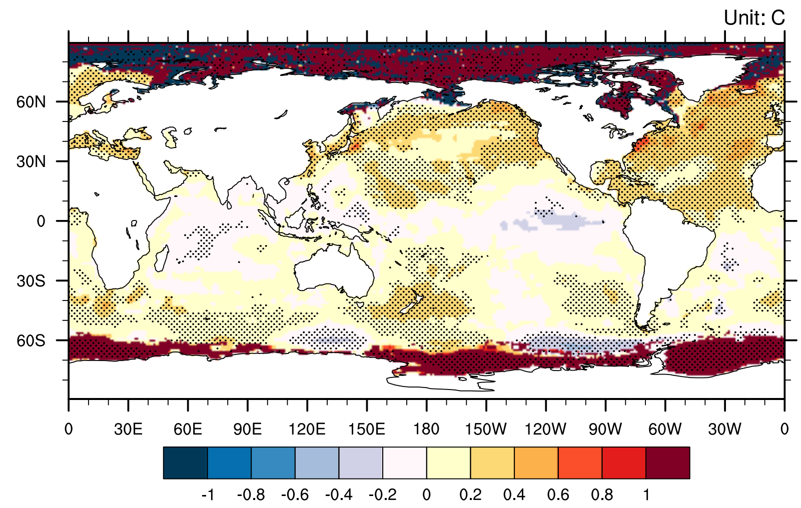

Fig. S4 The differences (unit: °C) of SST between silent volcano eruption period (1936-1962) and active volcano eruption periods (1901-1935 and 1963-1993) with linear trends removed

Stippling indicates the correlations are significant at the *p*=0.05 level based on student t-test.

Map was generated by NCAR Command Language (NCL).

The NCAR Command Language (Version 6.3.0) [Software]. (2016).

Boulder, Colorado: UCAR/NCAR/CISL/TDD. http://dx.doi.org/10.5065/D6WD3XH5

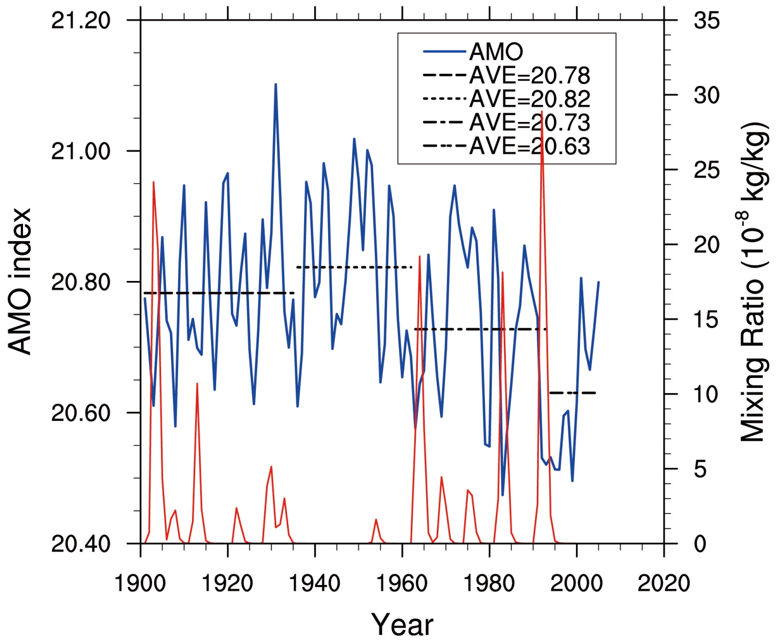

Fig. S5 The time series of simulated AMO index from the CESM1-CAM5 volcanic forcing sensitivity experiment

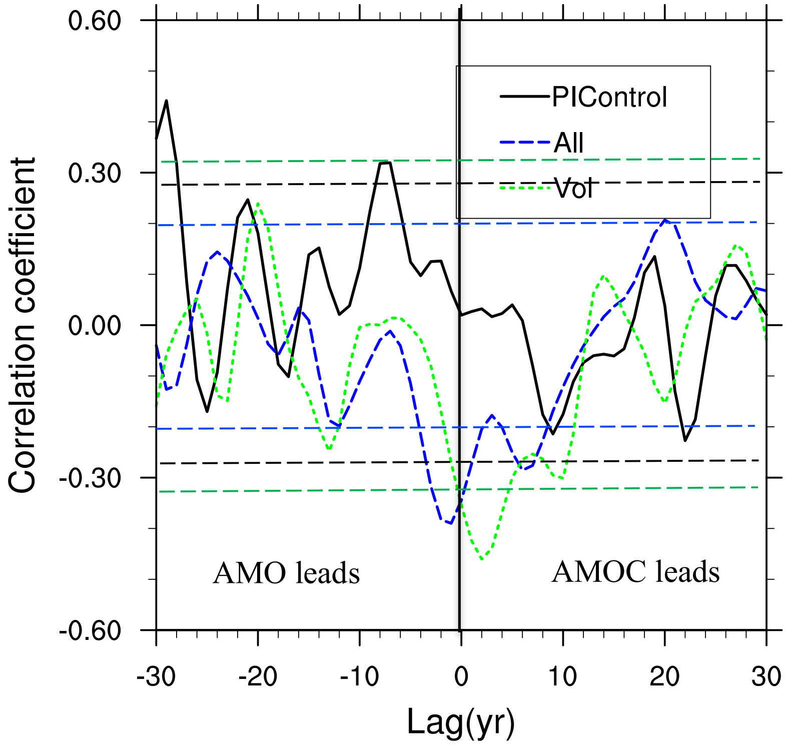

Fig. S6 The lag-correlations between AMOC and AMO indices from the CESM1-CAM5 PIControl experiment (black line), all-forcing experiment (blue line), and volcanic forcing experiment (green line)

Positive lags mean that the AMOC is leading. Negative lags mean that the AMO is leading. Black dash lines show the significance levels of PIControl correlation coefficients (p<0.1). Blue dash lines show the significance levels of all-forcing experiment correlation coefficients (p<0.1). Green dash lines show the significance levels of volcanic forcing experiment correlation coefficients (p<0.1).

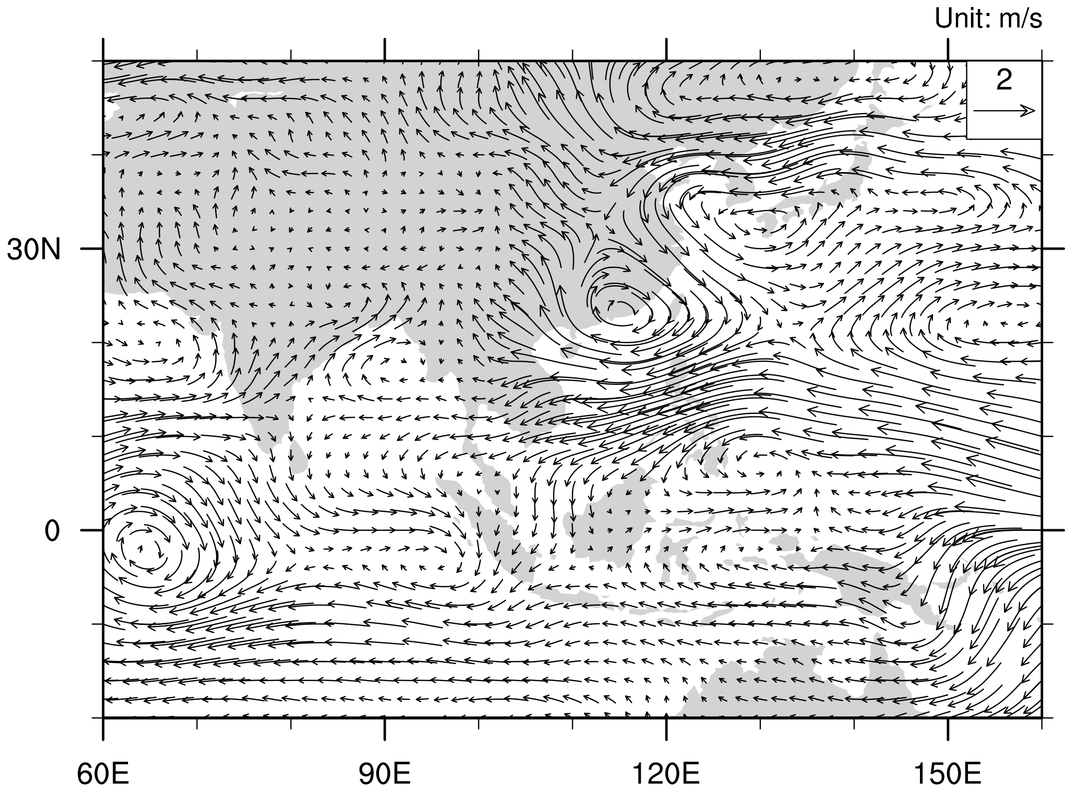

Fig. S7 The regression of AMO index on the summer 850hPa wind field (unit: m/s) over the Asian monsoon region

Map was generated by NCAR Command Language (NCL).

The NCAR Command Language (Version 6.3.0) [Software]. (2016).

Boulder, Colorado: UCAR/NCAR/CISL/TDD. http://dx.doi.org/10.5065/D6WD3XH5

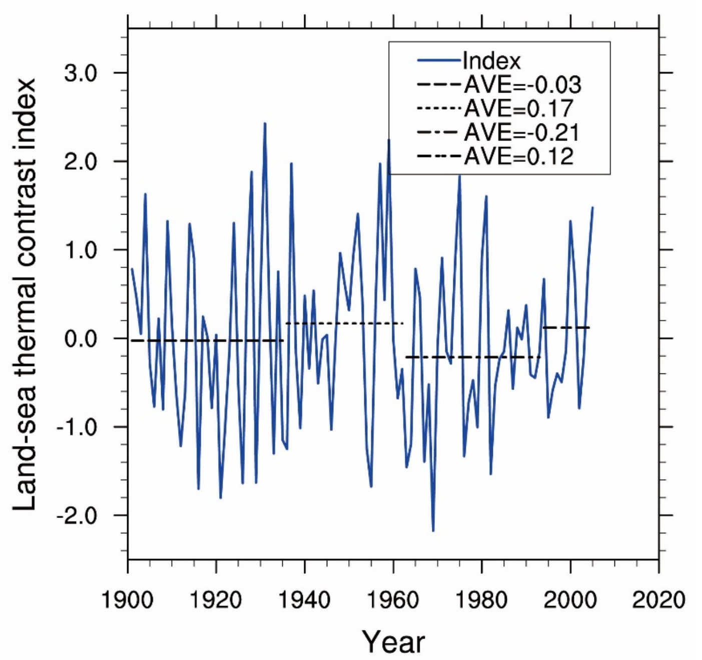

Fig. S8 The time series of simulated land-sea thermal contrast index from the CESM1-CAM5 volcanic forcing sensitivity experiment

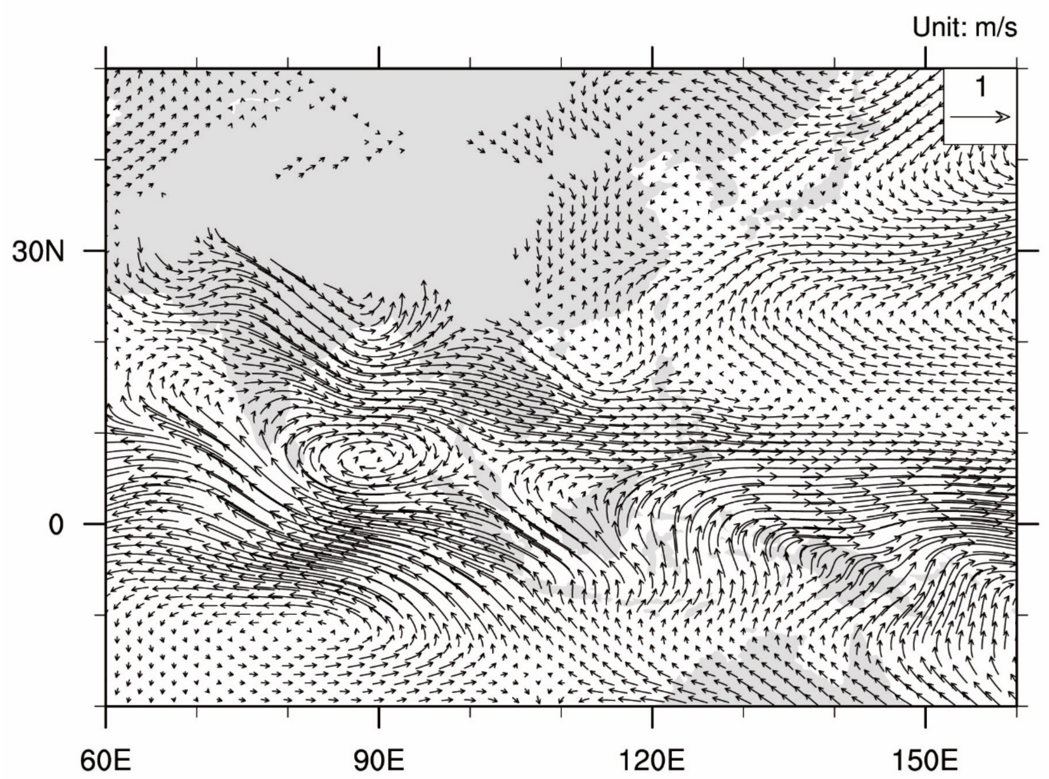

Fig. S9 The regression of land-sea thermal contrast index on the summer 850hPa wind field (unit: m/s) over the Asian monsoon region from the CESM1-CAM5 volcanic forcing sensitivity experiment

Map was generated by NCAR Command Language (NCL).

The NCAR Command Language (Version 6.3.0) [Software]. (2016).

Boulder, Colorado: UCAR/NCAR/CISL/TDD. http://dx.doi.org/10.5065/D6WD3XH5
